# Isolation and Characterization of *Phenylalanine Ammonia Lyase* (*PAL*) Genes in *Ferula pseudalliacea*: Insights into the Phenylpropanoid Pathway

**DOI:** 10.3390/genes15060771

**Published:** 2024-06-12

**Authors:** Pegah Shahidi, Bahman Bahramnejad, Yavar Vafaee, Dara Dastan, Parviz Heidari

**Affiliations:** 1 Department of Plant Production and Genetics, Faculty of Agriculture, University of Kurdistan, Sanandaj 6617715175, Iran; shahidi.pegah@gmail.com; 2 Department of Horticultural Sciences, Faculty of Agriculture, University of Kurdistan, Sanandaj 6617715175, Iran; y.vafaee@uok.ac.ir; 3 Department of Pharmacognosy, School of Pharmacy, Medicinal Plants and Natural Products Research Center, Hamadan University of Medical Sciences, Hamadan 6517838736, Iran; d.dastan@umsha.ac.ir; 4 Faculty of Agriculture, Shahrood University of Technology, Shahrood 3619995161, Iran

**Keywords:** phenylpropanoid pathway, cinnamate-4-hydroxylase, gene cloning, phenylalanine ammonia lyase

## Abstract

Phenylalanine ammonia lyase (PAL) is a key enzyme regulating the biosynthesis of the compounds of the phenylpropanoid pathway. This study aimed to isolate and characterize *PAL* genes from *Ferula pseudalliacea* Rech.f. (Apiales: Apiaceae) to better understand the regulation of metabolite production. Three *PAL* gene isoforms (*FpPAL1-3*) were identified and cloned using the 3′-RACE technique and confirmed by sequencing. Bioinformatics analysis revealed important structural features, such as phosphorylation sites, physicochemical properties, and evolutionary relationships. Expression analysis by qPCR demonstrated the differential transcription profiles of each *FpPAL* isoform across roots, stems, leaves, flowers, and seeds. *FpPAL1* showed the highest expression in stems, *FpPAL2* in roots and flowers, and *FpPAL3* in flowers. The presence of three isoforms of *PAL* in *F. pseudalliacea,* along with the diversity of *PAL* genes and their tissue-specific expression profiles, suggests that complex modes of regulation exist for phenylpropanoid biosynthesis in this important medicinal plant. The predicted interaction network revealed associations with key metabolic pathways, emphasizing the multifaceted roles of these *PAL* genes. In silico biochemical analyses revealed the hydrophilicity of the FpPAL isozyme; however, further analysis of substrate specificity and enzyme kinetics can clarify the specific role of each FpPAL isozyme. These comprehensive results increase the understanding of *PAL* genes in *F. pseudalliacea*, helping to characterize their contributions to secondary metabolite biosynthesis.

## 1. Introduction

Plants are considered the most accessible source of treatment in the traditional medicine and ancient health systems of various civilizations, where the use of herbs for medicinal purposes dates back as far as 4000 to 5000 BC [1]. Plants are valuable sources of a variety of biologically active compounds known as secondary metabolites that are used in different industries, including the food, cosmetic, and pharmaceutical industries, as well as in the manufacture of chemicals utilized in agriculture, such as herbicides and pesticides. Secondary metabolites encompass a diverse group of low-molecular-weight compounds that are employed by plants at low concentrations to create defense mechanisms against pathogens, pests, and abiotic stresses [2].

As the third most species-rich genus within the Apiaceae family (formerly known as Umbelliferae), *Ferula* comprises approximately 185 species with main geographical distributions in the Middle East, Mediterranean basin, and Eastern and Central Europe [3,4]. Among these, 30 species grow in Iran, 15 of which are endemic species [5]. For centuries, several species within this genus have been used in folk medicine to treat various diseases, such as gastrointestinal disorders, kidney stones, gallstones, asthma, bronchitis, diabetes, and rheumatism [6]. The major identified phytochemical constituents in the *Ferula* genus include coumarins, sesquiterpenes, sesquiterpene coumarins, and sesquiterpene lactones, which have documented biological activities, such as anticoagulant, antibacterial, antiviral, antiparasitic, antispasmodic, anti-inflammatory, and antitumor properties [3,4,6]. The discovery of new PAL isoforms in medicinal plants holds great potential for metabolic engineering applications. By utilizing these isoforms, researchers can enhance the production of compounds with medicinal properties in engineered microbial strains within industrial bioreactors [7]. This approach offers a sustainable and efficient method for the large-scale production of these valuable compounds [8,9]. The identification of *PAL* genes as promising candidates for future metabolite engineering projects further underscores the importance of this research in advancing biotechnological applications in the pharmaceutical industry.

*F. pseudalliacea* Rech.f. (Apiales: Apiaceae) is an indigenous species endemic to the western border of the Zagros Mountains in Kurdistan, western Iran, and is utilized in traditional folk medicine for different purposes, including killing human parasites and treating ulcers [10]. Currently, new disesquiterpene and sesquiterpene coumarins, including sanandajin, methyl galbanate, ethyl galbanate, fekrynol acetate, farnesiferol B, and kamonolol acetate, have been isolated and identified in *F. pseudalliacea* and found to have proven anticancer, antibacterial, and strong antioxidant effects [5,10]. Despite the presence of such valuable bioactive compounds, this species has a narrow geographical distribution with scattered populations and is therefore considered a threatened medicinal species [5].

The phenylalanine ammonia lyase (PAL) enzyme is a key enzyme that directs the metabolic flow from the primary to the secondary metabolism and more specifically to the phenylpropanoid part of it [11]. Given the important role of this pathway in the synthesis of a wide range of phenolic compounds with pivotal impacts on the protection of plants against biotic and abiotic stresses [12], as well as its role in plant growth and development, the *PAL* gene has been extensively studied by researchers in more than 1600 different plants belonging to 250 genera [13]. It seems that PALs are linked with diverse biological processes. For instance, it was reported that *PAL* genes are involved in “translocated” peach/plum graft incompatibility [14]. Moreover, most *PAL* genes in wheat showed upregulation in response to *Fusarium graminearum* infection [15]. The PAL gene family has a variable number of genes in different plant species, such as rice, which has nine members [16]; tomato, which has thirteen members [17]; potato, which has fifty members [18]; and most other plant species, which have four or five members [13].

The key genes involved in the phenylpropanoid biosynthesis pathway are regulated in a coordinated manner throughout plant growth and development, especially in response to biotic and abiotic stresses [19]. Elevating the level of phenolic compounds during stress conditions and fruit ripening is linked to an upsurge in the expression of genes that encode enzymes crucial for key reactions in the phenylpropanoid pathway, such as *PALs*. Although the sequence and overall function of *PAL* genes have already been explored in many plant species, little information is available on the sequence and expression pattern of *PALs* in industrially important and conversationally endangered *Ferula* species, particularly *F. pseudlliaceae*. The objective of the current study was to clone and sequence *PAL* gene isoforms from *F. pseudalliacea* (referred to as *FpPAL*) using the 3′-RACE technique. This was performed in order to determine the number of *PAL* isoforms present and to obtain their gene sequences. Additionally, bioinformatics analyses were conducted on the FpPAL sequences to identify conserved domains, evolutionary relationships, and predicted structural features. Furthermore, the study aimed to quantify the expression profiles of each *FpPAL* isoform in the roots, stems, leaves, flowers, and seeds of *F. pseudalliacea* plants using qPCR. This was performed to gain insight into the activity of these isoforms in different organs.

## 2. Materials and Methods

### 2.1. Plant Material

**Figure 1:**
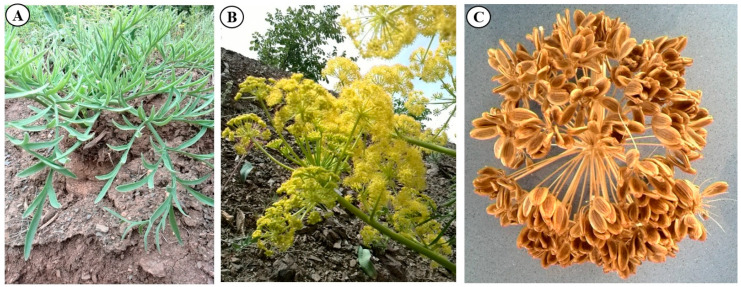
Morphology of *F. pseudalliaceae*. (**A**) Rosset growth of young plants; (**B**) inflorescence at the full blooming stage; (**C**) fully ripened seeds.

Different parts of *F. pseudalliacea*, including the roots, stems, leaves, flowers, and seeds, were collected from uniform, well-grown plants (Figure 1) from their natural niche in Gazne village (10 km west of Sanandaj), Iran (35°08′38.1″ N 46°57′45.4″ E). The collected samples were immediately frozen in liquid nitrogen and stored at −80 °C until analysis. Botanical identification of the *F. pseudalliacea* was performed by Eng. Hossein Hossein Maroofi (Agriculture and Natural Resources Research Center), and a voucher sample was deposited in the herbarium housed at the Faculty of Agriculture, University of Kurdistan, Iran.

### 2.2. DNA and RNA Extraction and cDNA Synthesis

Total genomic DNA was extracted from leaf samples according to the CTAB method, with minor modifications [20]. RNA was eliminated from purified DNA samples by treatment with DNase-free ribonuclease A for 1 h at 37 °C (34390, SERVA, Heidelberg, Germany). Total RNA was extracted from frozen tissue from the roots, stems, leaves, flowers, and seeds according to previous methods [21]. The RNA samples were treated with DNase I (Pishgam Biotech, Tehran, Iran) to remove DNA. The quantity and quality of the extracted DNA and RNA samples were evaluated using both a NanoDrop device (Thermo Scientific™ NanoDrop™ OneC, Waltham, MA, USA) and agarose gel electrophoresis. First-strand cDNA was synthesized from 1 μg of the purified RNA sample using a commercially available reverse transcriptase kit (Yekta Tajhiz Azma Co., Tehran, Iran) according to the manufacturer’s instructions.

### 2.3. Primer Design and Gene Cloning and Sequencing

Due to the lack of genomic resources for *F. pseudalliacea*, the designing of PAL primers was based on conserved regions of the gene for which sequence information was available. Actin was used as a reference gene and primers were designed based on sequences of closely related relatives of the same Apiaceae family, including *Daucus carota* L., *Angelica gigas* Engelwortel., *Peucedanum praeruptorum* Dunn., and *Petroselinum crispum* Mill. Alignments were performed with Clustal Omega [22] to identify conserved proteins, which were subsequently used to design degenerate primers with Primer3 [23]. The primers used were analyzed in silico to avoid self-dimers, hairpins, and mispriming via the Oligo Analyzer online software v. 3.1 (http://eu.idtdna.com, accessed on 10 February 2024). Initial PCR was used to amplify a *PAL* core fragment using PAL-F and PAL-R primers (resulting in a 900 bp fragment), which was subsequently extended by 3’-RACE to obtain complete gene termini. At first, after RNA extraction, Oligo (dT) primer was used in the cDNA synthesis stage to obtain the complete gene. Then, using F-PAL2-D and PCR anchor primers (Table 1), the end region of the desired gene was amplified, resulting in a 700 bp fragment. The amplified products representing putative *FpPAL* genes were first purified from gels using an N0041101 Gene MARK Kit (BioElegen Tech, Taichung, Taiwan), then cloned and inserted into the pTG19-T vector using TA cloning, and subsequently transformed into *E. coli* DH5α chemically competent cells via the heat-shock method. Transformed colonies were screened by blue–white selection, and transformations were confirmed via colony PCR with the universal primer M13 and PAL-specific primers [24]. Plasmids from 10 positive clones per transformation were sequenced bidirectionally by Sanger sequencing (Applied Biosystems, Foster City, CA, USA), and the sequences were subsequently edited using Geneious v.1 software and registered in the NCBI database under accession numbers MH987773.1 (*FpPAL1*), MH987774.1 (*FpPAL2*), and MH987775.1 (*FpPAL3*). The resulting FpPAL sequences were confirmed via BLASTx homology searches against the NCBI nr database.

### 2.4. Phylogeny Analysis

The sequences of FpPAL proteins, along with their known orthologs in *Arabidopsis*, rice (*Oryza sativa*), carrot (*D. carota)*, sunflower (*Helianthus annuus)*, rapeseed (*Brassica napus*), camelina (*Camelina sativa*), and *Brachypodium distachyon*, were analyzed to investigate their evolutionary relationships. First, all the sequences were aligned using multiple alignments of the Clustal Omega tool [22], and the alignment output was submitted to the IQ tree online tool [25] to construct a phylogenetic tree using the maximum likelihood method with 1000 bootstraps. Finally, a tree of PAL family members was constructed using the iTOL online tool [26]. In addition, InterProscan online analysis [27] was used to identify the conserved domain in the FpPAL proteins.

### 2.5. Prediction of FpPAL Phosphorylation Regions and Interaction Network

To predict the phosphorylation sites in FpPAL proteins, the NetPhos [28] was used, for which the potential was set at ≥0.90. Additionally, an interaction network of FpPALs was constructed using the String database [29], based on available information from the dicot model plant *Arabidopsis*. In addition, significant KEGG pathways (FDR ≤ 0.05) linked to network nodes were identified. Moreover, the physicochemical properties of FpPAL proteins were predicted by the ProtParam tool [30].

### 2.6. Real-Time PCR Analysis

The expression levels of the three cloned PAL *(FpPAL 1-3*) isoforms were analyzed via quantitative real-time PCR (qPCR) for comparison across different *F. pseudalliacea* tissues. The reactions used cDNA templates synthesized from roots, stems, leaves, flowers, and seeds with gene-specific primers designed from 3′-UTR regions. The *FpACTIN* housekeeping gene was used as an internal control. The qPCR reactions were carried out in three biological and two technical replications in 15 μL reactions using SYBR Green Master Mix (Yekta Tajhiz Azma, Tehran, Iran) on an ABI StepOne Plus system (Life Technologies, Carlsbad, CA, USA). Cycling conditions were 95 °C for 3 min, followed by 40 cycles at 95 °C for 20 s and at 60 °C for 30 s. Melting curve analysis confirmed single peak amplification. Relative expression levels were calculated by the ΔΔCt method [31] using *FpACTIN* for normalization and then calibrating the expression against the lowest expressing tissue. Statistical analysis was performed via one-way ANOVA with Tukey’s post hoc test in SPSS v.22 software.

## 3. Results

### 3.1. Screening and Confirmation of Clones

**Figure 2:**
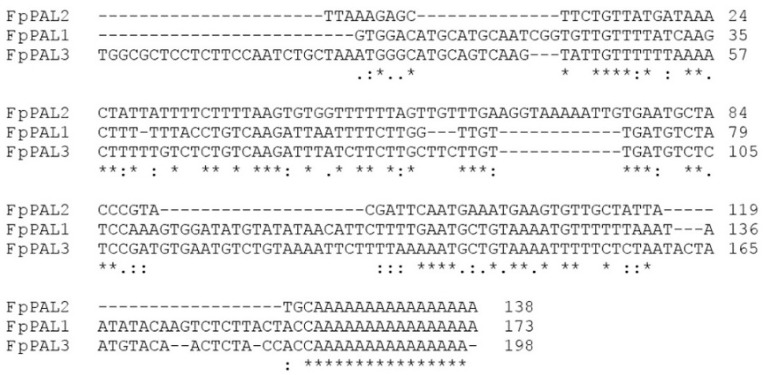
Multiple-alignment analysis of the 3’ UTRs of *FpPAL1*, *FpPAL2*, and *FpPAL3*. Regions of 3’ UTRs of the *FpPAL* isoforms were amplified using 3’-RACE method. * shows positions that have a single, fully conserved residue between *FpPALs*. Colon and dot symbols indicate conserved substitutions and semi-conserved substitutions, respectively.

To clone the *FpPAL* genes, the respective amplified fragments were gel-purified and inserted into an empty PTG-19 vector. Modified vectors were transformed into the *E. coli* strain TOP 10 and sequences were validated with Sanger sequencing. After 16–20 h of bacterial culture in culture medium containing the antibiotic ampicillin, some of the grown clones were selected (white clones containing recombinant plasmid) and cultivated in new medium. A colony PCR test was performed using specific primers for each gene and the M13 universal primer on selected single clones. The agarose gel electrophoresis product of the PCR confirmed the success of the work, and the 3′ region of the *FpPAL* genes was also amplified. The obtained sequences were blasted against the NCBI database, and PAL sequences were confirmed. The BLAST results showed that the three isoforms of the *FpPAL* gene are most similar to those of the *D. carota* genes. Finally, the sequences were registered in the NCBI database (MH987773, MH987774, and MH987775), for the three members of the *FpPAL* gene. Alignment of the 3’-UTRs of the three *FpPAL* gene isoforms revealed that this region has high diversity (Figure 2 and Figure S1). In this region, the stop codon in the FpPAL1 sequence was located at nucleotide 173, and that in the FpPAL2 and FpPAL3 sequences was located at nucleotides 138 and 198, respectively. Due to the lack of *F. pseudaliacea* genome sequence data, the sequence of the 3’-UTR was used to design specific primers for each *FpPAL* gene isoform to investigate their expression.

### 3.2. Structure and Physicochemical Analysis of FpPALs

**Figure 3:**
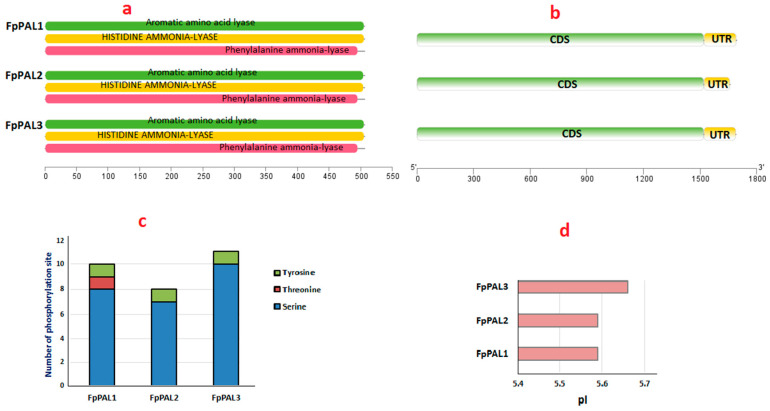
Analysis of the three phenylalanine ammonia lyase genes to find conserved domains (**a**) and gene structure (**b**); prediction of serine, threonine, and tyrosine phosphorylation sites (**c**); estimation of isoelectric points (pIs) (**d**) of the isolated FpPALs.

The protein sequence analysis of FpPALs revealed that all three proteins (Figure 3a and Figure S2) belong to the aromatic amino acid lyase group (IPR001106), which has two subgroups, histidine ammonia lyase (HAL) and phenylalanine ammonia lyase (Figure 3a and Figure S2). Furthermore, all *FpPALs* share a common gene structure (Figure 3b). Phosphorylation is among the post-translational modifications that play an important role in regulating the activity and lifespan of proteins. In this study, FpPAL proteins were compared with each other in order to develop a better understanding of how they are affected by phosphorylation (Figure 3c). The results revealed that FpPAL3 has more potential sites for phosphorylation than FpPAL1 and FpPAL2. In addition, the physicochemical properties of FpPALs were estimated. Our findings showed that all the studied proteins had a pI less than 6.0 (Figure 3d) and a negative GRAVY value, suggesting that they are hydrophilic proteins and can be activated under acidic conditions.

### 3.3. Phylogenetic Analyses

**Figure 4:**
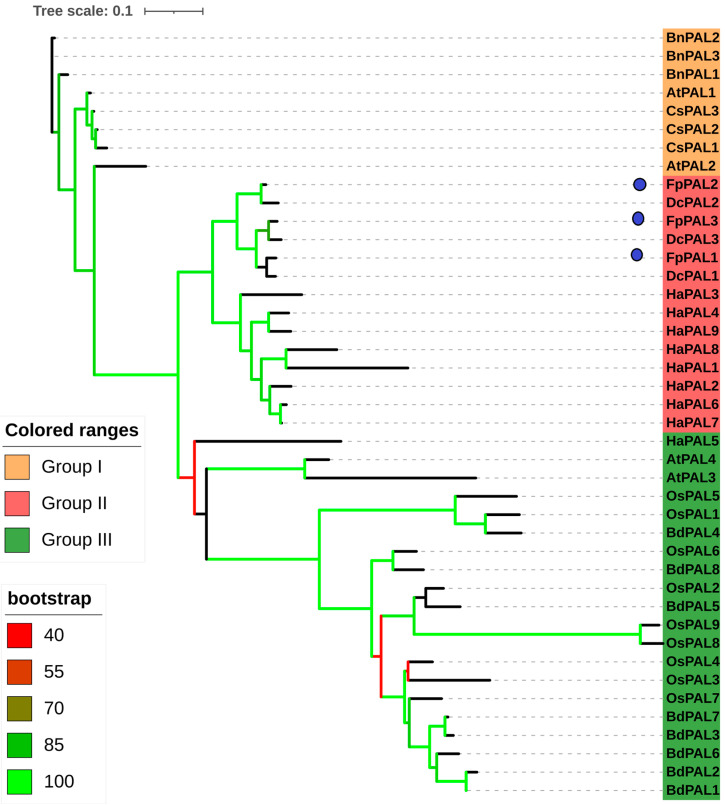
Phylogeny analysis of PALs using the maximum likelihood method. Genes from *Arabidopsis* (starting with At), *O. sativa* (starting with Os), *C. sativa* (starting with Cs), *B. distachyon* (starting with Bd), *B. napus* (starting with Bn), *D. carota* (starting with Dc), and *H. annuus* (starting with Ha) were investigated.

To study the evolutionary relationships between *FpPALs* and their orthologs from other plants, a phylogenetic tree was constructed based on the maximum likelihood method (Figure 4). All PAL proteins were classified into three main groups. The cloned FpPAL genes and their orthologs from *D. carota* and *H. annuus* were located in group 2. Moreover, the PALs from monocot plants were more genetically distant from than orthologous to the PALs from dicotyledons species. These results indicate that the diversity of the members of this gene family occurred after the separation of monocots and dicots.

### 3.4. Interaction Analysis

**Figure 5:**
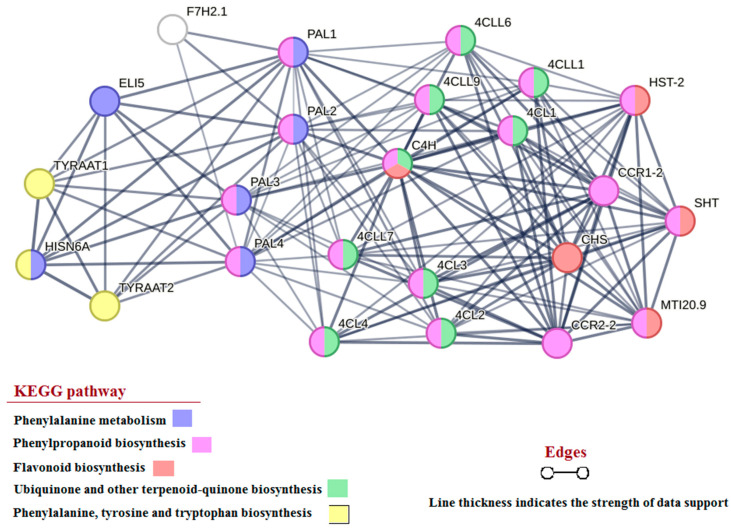
Interaction analysis of PAL proteins. Graph showing the interaction of PAL proteins with other proteins of the *A. thaliana* metabolic network. CCR: Cinnamoyl-CoA reductase; 4CL: 4-coumarate-CoA ligase; CHS: chalcone synthase; HST: shikimate O-hydroxycinnamoyltransferase; MTI20.9: HXXXD-type acyl-transferase family protein; SHT: spermidine hydroxycinnamoyl transferase; 4CLL9: 4-coumarate-CoA ligase-like 9; ELI5: Tyrosine decarboxylase 1; F7H2.1: F-box/kelch-repeat protein; HISN6A: histidinol-phosphate aminotransferase 1; TYRAAT2: arogenate dehydrogenase 2.

An interaction network for FpPALs was constructed based on available data from the dicot model plant *Arabidopsis thaliana* (Figure 5). Results revealed that PAL proteins strongly interact with proteins involved in phenylalanine metabolism, phenylpropanoid biosynthesis, flavonoid biosynthesis, ubiquinone and other terpenoid-quinone biosynthesis, phenylalanine biosynthesis, and tyrosine and tryptophan biosynthesis pathways. In addition, C4H was predicted to be a key point in the network that participates in different pathways and interacts with different proteins. PALs exhibited unique interactions with the HisN6A (histidinol-phosphate aminotransferase) and TYRAAT (arogenate dehydrogenase) proteins, which are involved in the phenylalanine, tyrosine, and tryptophan biosynthesis pathways. PALs are involved in various cellular pathways, and thus probably have multiple interactions directly or indirectly with other key elements of cellular pathways.

### 3.5. Expression Analysis

**Figure 6:**
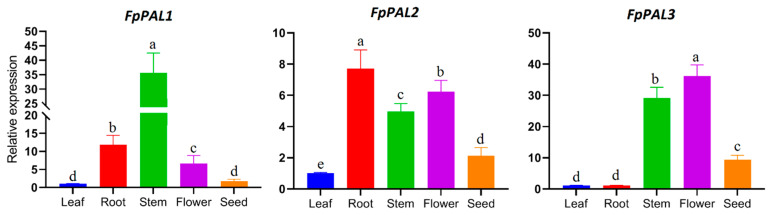
Expression analysis of *FpPAL* genes in different tissues (leaf, root, stem, flower, seed) of *F. pseudalliacea.* Different letters on the means represent statistically significant differences, as determined by Tukey’s test, at a significance level of *p* < 0.05.

The expression patterns of *FpPAL* genes were investigated in different tissues (Figure 6). The three *FpPAL* genes exhibited diverse expression patterns in different tissues of *F. pseudalliacea*. They, generally, had very low expression levels in the leaves compared to other tissues. That is why the expression in the leaves was set as the basal expression level to which the expression from the rest of the tissues was compared. The expression level of *FpPAL1* significantly increased in the stem, while its expression was less induced in the seeds. In contrast, *FpPAL2* showed high expression levels in both root tissues and flowers. *FpPAL3* exhibited higher expression in floral and stem tissues, with less induction in roots. These diverse expression patterns of *FpPAL* genes suggest tissue-specific expression. Considering that PALs are involved in different pathways, it is possible that each isoform of this gene family has received a more specific function, which has led to tissue-specific expression.

## 4. Discussion

Phenylpropanoids are important active plant compounds that participate in the production of valuable secondary metabolites. The PAL enzyme is a main component of the phenylpropanoid biosynthesis pathway. In addition, *PAL* genes are regulated in a coordinated manner during plant growth and development, particularly under biotic and abiotic stresses [13,19]. The members of the PAL family vary widely in their numbers among species. For example, there are nine PAL genes in rice [16], thirteen genes in the tomato [17], fourteen genes in the potato [32], thirty-seven genes in common wheat [33], twelve genes in the walnut [34], four genes in the Hainan plum-yew [35], fifteen genes in the grape [36], and seven genes in the cucumber [37]. However, most diploid plants have four or five PAL family members [38,39,40]. In the present study, three members of the PAL family in *F. pseudaliacea* (*FpPALs*) were identified and cloned. The number of PAL family members may be associated with the metabolic diversity and ecological niche of various plant species, offering valuable insights into their characteristics. They appeared, most probably, by duplication events that could further affect the expansion of the PAL gene family [41]. A low number of exons in a gene can shorten the gene-editing process, increasing the speed of gene expression [42]. It seems that *FpPAL* genes are classified as early response genes, although additional studies are needed. It was also predicted that FpPALs are hydrophilic proteins that are more active in a slightly acidic environment. These characteristics are useful in understanding the engineering of the metabolic pathways in which the FpPAL enzyme is involved. Phylogenetic analysis revealed that *FpPALs* have evolutionary relationships with *PALs* from *D. carota* and *H. annuus* and that they have a high genetic distance from PALs from monocots, indicating that they have undergone distinct evolutionary processes [43].

A potential interaction network of FpPALs revealed that these enzymes are directly/indirectly involved in several key metabolic pathways, such as phenylalanine metabolism, phenylpropanoid biosynthesis, flavonoid biosynthesis, ubiquinone and other terpenoid-quinone biosynthesis, phenylalanine, and tyrosine and tryptophan biosynthesis pathways. By harnessing these isoforms, researchers have the ability to boost the production of compounds with medicinal properties in engineered microbial strains housed within industrial bioreactors. This innovative approach provides a sustainable and efficient method for the large-scale production of these highly sought-after compounds. The PAL enzyme is known as the turning point of primary and secondary metabolites and is the first enzyme involved in the phenylpropanoid pathway [13,40]. The phenylpropanoid pathway plays a key role in the response to a wide range of phenolic compounds and has pivotal impacts on the protection of plants against biotic and abiotic stresses [12], as well as on plant growth and development [13]. 

The *FpPAL* gene exhibited diverse expression patterns in different tissues of *F. pseudalliacea*. The tissue-specific expression of *FpPAL* genes may contribute to the biosynthesis of specific phenolic compounds in different tissues and their role in plant defense and adaptation to environmental challenges. Tissue-specific differences in PAL gene expression have also been reported in previous studies. For example, *PAL5* has no detectable transcript level in the stem of tomato plants [44]. A review of the literature reveals that the activity of PAL is tightly regulated at the transcriptional level by developmental stages. This phenomenon is attributed to variations in PAL activity that are linked to the diverse functions played by its various secondary metabolites in processes such as plant development and tolerance to abiotic stressors [45,46,47]. In addition, the overlapping and diverse expression patterns of *PAL* gene isoforms in watermelon have been reported [48]; also, these differences in *PAL* gene transcription levels have been observed in the roots, stems, and leaves of ginseng plants [49]. This difference in expression patterns may be related to differences in the upstream regulatory systems of these genes and their possible interactions [42]. Additionally, these results reveal the different roles of these genes during the stages of plant growth and development. The high expression level of *FpPALs* in the inflorescence of *F. pseudalliacea* may indicate the amount of compounds that these genes affect by playing a role in their biosynthesis, as in *Arabidopsis*. There is a high expression of the *PAL* gene in *Arabidopsis* flowers during the period of flower growth, where the increase in *PAL* expression has been associated with an increase in phenylpropanoid compounds such as sinapate and various flavonoids [50]. The amount of total phenolic compounds in relation to *PAL* gene expression in different organs of *F. pseudalliacea* was also reported [5]. The highest amount of phenolic compounds was detected in the methanolic extract of flowers cut with ethyl acetate, followed by the methanolic extract of unripe seeds cut with ethyl acetate. 

In our study, *FpPAL3* had the highest expression level in floral and seed organs compared to the expression of *FpPAL1* and *FpPAL2*. The transcription level of this gene in flowers was approximately six times greater than that in immature seeds, which was approximately four times greater than the expression level in leaves. Considering the expression levels of the *FpPAL3* gene, it can be concluded that this gene may play a more important role in the production of phenolic compounds in flowers and immature seeds than its other two isoforms.

## 5. Conclusions

In this study, the *phenylalanine ammonia lyase* (PAL) gene was successfully isolated and cloned from *F. pseudalliacea* for the first time. Through the innovative 3’-RACE technique, three distinct isoforms of this gene were identified. Surprisingly, these isoforms shared a strikingly high degree of sequence similarity, suggesting potential duplication events in their evolutionary history. Despite their structural similarities, the expression patterns of these genes varied significantly across different tissues. This divergence in expression hints at the presence of tissue-specific regulatory mechanisms. Notably, the *FpPAL3* gene displayed the highest expression levels in floral tissues. Given the abundance of phenolic compounds in these tissues, it is plausible to infer that *FpPAL3* plays a pivotal role in the biosynthesis of valuable metabolites in *F. pseudalliacea* flowers. While these findings shed light on the potential functions of *PAL* genes in *F. pseudalliacea*, further research is warranted to fully characterize the enzymatic properties of these isoforms. The development of transgenic lines harboring *PAL* genes and the engineering of PAL-related metabolites in bacterial strains hold immense promise for future biotechnological applications. Such endeavors could pave the way for novel approaches in the production of valuable compounds with diverse industrial applications.

## Figures and Tables

**Table 1:** List of primers used in the present study to clone *PAL* gene isoforms and qPCR analyses in *F. pseudalliacea* tissues. F: forward primer; R: reverse primer; RT: designates the primers for the quantitative real-time PCR.

Primer Name	Sequence (5′-3′)	tm °C	Product Size
F-PAL	TACATYGCTGGACTTCTMACTGG	58	960
R-PAL	CTCCTCCAARTGCCTCARGTC		
F-PAL2-D	GTCCAAAGYGCTGARCARCAC	60	860
Oligo(dT) primer	GACCACGCGTATCGATGTCGACTTTTTTTTTTTTTTTTV		
PCR anchor primer	GACCACGCGTATCGATGTCGAC		
F-FpPAL1.RT	CACAGGAGAAAAAGTGCGGTCA	60	179
R-FpPAL1.RT	CTTGATAAAACAACACCGATTGC		
F-FpPAL2.RT	CATAAGGGAAGAGTTGGGG	60	182
R-FpPAL2.RT	CATAACAGAAGCTCTTTAATTAAGCA		
F-FpPAL3.RT	CAGAACTGGGAACCGAATATCTT	60	175
R-FpPAL3.RT	ATACTTGACTGCATGCCCATTTAG		
F-FpACTIN.RT	GCCATCTATGATTGGGAATGG	56	190
R-FpACTIN.RT	GCCACCACCTTGATCTTCATG		

## Data Availability

The data generated or analyzed in this study are included in this article. Other materials that support the findings of this study are available from the corresponding author upon reasonable request.

## Supplementary Materials

The following supporting information can be downloaded at: https://www.mdpi.com/article/10.3390/genes15060771/s1, Figure S1: Nucleotide sequence alignment of *PAL1*, *PAL2*, and *PAL3* genes from *F. pseudalliacea*. In the Consensus sequence, the letter N indicates a variation in each of the three sequences; Figure S2: Protein sequence alignment of *PAL1*, *PAL2*, and *PAL3* genes from *F. pseudalliacea*. In the Consensus sequence, the letter N indicates a variation in each of the three sequences.
