# Supplementary material for: Isolation and Characterization of Phenylalanine Ammonia Lyase (PAL) Genes in Ferula pseudalliacea: Insights into the Phenylpropanoid Pathway
Source: Genes (Basel). 2024 Jun 12;15(6):771. doi: 10.3390/genes15060771 (PMC11203166; doi:10.3390/genes15060771)
Supplement: Supplementary file 1 [file genes-15-00771-s001.zip › Figure S1.pdf]

```
F.pse PAL1  TACATCGCTG GACTTCTCAC TGGCCGTCCC AATTCTAAGG CGGTTGGACC CACTGGAGAA 60
F.pse PAL3  TACATCGCTG GACTTCTAAC TGGCCGCCCC AATTCCAAGG CTGTTGGACC CACTGGAGTA 60
F.pse PAL2  TACATTGCTG GACTTCTAAC TGGCCGTCCC AATTCTAAGG CGGTTGGACC CACTGGAGAA 60
 Consensus  TACATCGCTG GACTTCTAAC TGGCCGTCCC AATTCTAAGG CGGTTGGACC CACTGGAGAA

F.pse PAL1  AACCTGACAG CTGAAGAAGC GTTTAAACTT GCTGGAGTTG AAGGAGGATT TTTCGAGTTG 120
F.pse PAL3  ACTCTCAGCC CCGAAGAAGC ATTTAAGCTT GCTGGAGTTG AAGGTGGATT TTTCGAGTTA 120
F.pse PAL2  AACCTGACAG CTGAAGAAGC TTTTAAACTT GCTGGAGTTG ATGGTGCATT CTTTGAGCTT 120
 Consensus  AACCTGACAG CTGAAGAAGC NTTTAAACTT GCTGGAGTTG AAGGTGGATT TTTCGAGTTN

F.pse PAL1  CAGCCAAAAG AAGGCCTGGC ACTTGTTAAT GGAACAGCTG TTGGTTCTGG CATGGCCTCA 180
F.pse PAL3  CAGCCAAAAG AAGGCCTAGC ACTTGTTAAT GGAACAGCTG TTGGTTCTGG CATGGCCTCT 180
F.pse PAL2  CAGCCAAAAG AAGGCCTTGC GTTAGTCAAT GGCACAGCCG TTGGTTCTGG GATGGCTTCG 180
 Consensus  CAGCCAAAAG AAGGCCTNGC ACTTGTTAAT GGAACAGCTG TTGGTTCTGG CATGGCCTCN

F.pse PAL1  ATGGTACTTT TTGAGGCTAA TATATTAGCC GTTTTAGCCG AAGTTATGTC AGCAATTTTC 240
F.pse PAL3  ATGGTACTTT TTGAGGCTAA TATATTAGCA GTTCTATCTG AAGTTATGTC AGCAATTTTC 240
F.pse PAL2  ATGGTTCTGT TTGAAGCCAA TATACTTGCT GTGTTAGCTG AGGTTATGTC AGCTATATTT 240
 Consensus  ATGGTACTTT TTGAGGCTAA TATATTAGCN GTTTTAGCTG AAGTTATGTC AGCAATTTTC

F.pse PAL1  GCTGAAGTGA TGCAAGGAAA GCCTGAATTC ACAGACCATT TGACACATAA GTTGAAACAC 300
F.pse PAL3  GCTGAAGTGA TGCAAGGGAA GCCTGAATTT ACCGACCATT CGACACATAA GTTGAAGCAC 300
F.pse PAL2  GCTGAAGTGA TGCAGGGCAA ACCAGAATTC ACTGACCATT TGACACATAA GTTGAAGCAT 300
 Consensus  GCTGAAGTGA TGCAAGGNAA GCCTGAATTC ACNGACCATT TGACACATAA GTTGAAGCAC

F.pse PAL1  CATCCGGGCC AAATTGAGGC TGCGGCTATA ATGGAACACA TTTTGGATGG AAGTGCTTAT 360
F.pse PAL3  CATCCAGGGC AGATTGAGGC TGCAGCTATA ATGGAACACA TCTTGGATGG AAGCGCTTAC 360
F.pse PAL2  CATCCTGGGC AAATTGAAGC TGCAGCTATA ATGGAACACA TTTTGGATGG AAGCTCTTAT 360
 Consensus  CATCCNGGGC AAATTGAGGC TGCAGCTATA ATGGAACACA TTTTGGATGG AAGCGCTTAT

F.pse PAL1  GTTAAGGCTG CTCAGAAGCT ACATGAAATG GATCCATTAC AAAAACCAAA ACAGGACAGA 420
F.pse PAL3  GTTAAGGCTG CTCAGAAGCT CCATGAAATG GATCCATTAC AAAAACCAAA ACAAGACAGA 420
F.pse PAL2  GTTAAGGCTG CTCAGAAGCA ACATGAAATG GATCCCTTGC AAAAACCAAA GCAAGATAGA 420
 Consensus  GTTAAGGCTG CTCAGAAGCT ACATGAAATG GATCCATTAC AAAAACCAAA ACAAGACAGA

F.pse PAL1  TATGCTCTTA GAACATCTCC TCAGTGGCTT GGTCCTCAAA TTGAAGTGAT TCGATCATCG 480
F.pse PAL3  TATGCTCTTA GAACATCTCC TCAATGGCTA GGTCCTCAAA TTGAAGTGAT CAGATCATCG 480
F.pse PAL2  TATGCTCTTA GGACATCTCC ACAATGGCTT GGACCACAGA TTGAAGTGAT TCGATCGTCA 480
 Consensus  TATGCTCTTA GAACATCTCC TCAATGGCTT GGTCCTCAAA TTGAAGTGAT TCGATCATCG

F.pse PAL1  ACTAAAATGA TCGAAAGAGA GATTAACTCA GTCAATGATA ACCCATTAAT TGATGTTTCC 540
F.pse PAL3  ACTAAAATGA TCGAAAGAGA AATCAACTCT GTCAACGATA ACCCATTGAT TGATGTTTCC 540
F.pse PAL2  ACAAAAATGA TAGAAAGGGA GATCAATTCT GTCAATGATA ACCCTTTGAT TGATGTGTCG 540
 Consensus  ACTAAAATGA TCGAAAGAGA GATCAACTCT GTCAATGATA ACCCATTGAT TGATGTTTCC

F.pse PAL1  AGGAACAAAG CTATACATGG TGGCAATTTC CAGGGCACAC CTATTGGAGT GTCAATGGAC 600
F.pse PAL3  AGGAACAAGG CTATACATGG TGGAAATTTC CAGGGCACCC CAATTGGTGT TTCGATGGAC 600
F.pse PAL2  AGGAACAAGG CCATACATGG TGGTAATTTC CAGGGTACCC CTATTGGAGT TTCTATGGAC 600
 Consensus  AGGAACAAGG CTATACATGG TGGNAATTTC CAGGGCACCC CTATTGGAGT TTCNATGGAC

F.pse PAL1  AATACACGTC TGGCTATTGC AGCAATAGGA AAGCTCATGT TTGCTCAATT TTCAGAACTT 660
F.pse PAL3  AACACACGTT TGGCTATTGC AGCCATTGGA AAGCTGATGT TTGCTCAGTT TTCAGAACTT 660
F.pse PAL2  AATACAAGGT TGGCTATTGC AGCAATTGGA AAGCTCATGT TCGCCCAATT TTCGGAGCTT 660
 Consensus  AATACACGTT TGGCTATTGC AGCAATTGGA AAGCTCATGT TTGCTCAATT TTCAGAACTT

F.pse PAL1  GTCAACGATT TTTACAACAA TGGGTTGCCA TCAAATTTGT CTGGAGGGCG TAATCCAAGT 720
F.pse PAL3  GTCAACGATT TTTACAACAA TGGGTTGCCA TCCAATTTGT CTGGAGGGCG TAACCCAAGC 720
F.pse PAL2  GTGAATGACT TTTATAACAA TGGGCTGCCA TCTAATCTTT CTGGAGGGCG TAATCCGAGT 720
 Consensus  GTCAACGATT TTTACAACAA TGGGTTGCCA TCNAATTTGT CTGGAGGGCG TAATCCAAGT

F.pse PAL1  TTGGATTATG GATTCAAGGG AGCTGAGATT GCCATGGCTT CTTACTGCTC TGAACTTCAA 780
F.pse PAL3  TTGGATTATG GATTCAAGGG TGCTGAAATT GCCATGGCAT CATACTGCTC TGAACTACAG 780
F.pse PAL2  TTGGATTATG GTTTTAAGGG CGCTGAAATT GCTATGGCTT CTTACTGCTC CGAGCTACAA 780
 Consensus  TTGGATTATG GATTCAAGGG NGCTGAAATT GCCATGGCTT CTTACTGCTC TGAACTACAA

F.pse PAL1  TTTTTAGCCA ATCCAGTGAC CAACCATGTC CAAAGCGCTG AGCAACACAA TCAAGATGTC 840
F.pse PAL3  TTTTTAGCCA ATCCAGTGAC TAACCATGTC CAAAGCGCTG AGCAGCACAA TCAAGATGTG 840
F.pse PAL2  TTTTTAGGCA ATCCAGTTAC TAACCATGTC CAAAGCGCTG AACAACACAA TCAAGATGTG 840
 Consensus  TTTTTAGCCA ATCCAGTGAC TAACCATGTC CAAAGCGCTG AGCAACACAA TCAAGATGTG
```

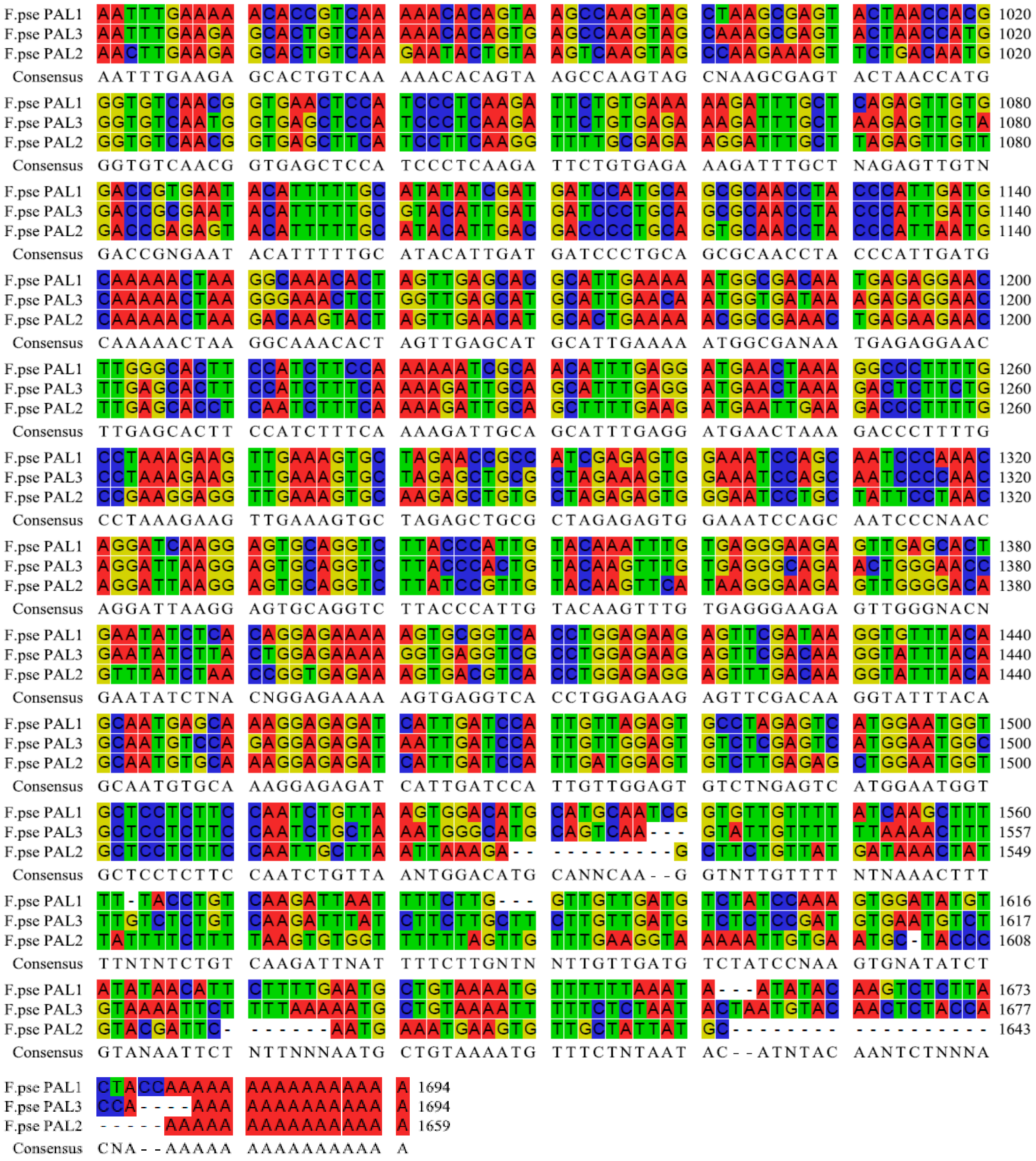

Figure S1. Nucleotide sequences alignment of *PAL1, PAL2*, and *PAL3* genes from *Ferula pseudalliacea*. In the Consensus sequence, the letter N indicates a variation in each of the three sequences.
