# Supplementary material for: Isolation and Characterization of Phenylalanine Ammonia Lyase (PAL) Genes in Ferula pseudalliacea: Insights into the Phenylpropanoid Pathway
Source: Genes (Basel). 2024 Jun 12;15(6):771. doi: 10.3390/genes15060771 (PMC11203166; doi:10.3390/genes15060771)
Supplement: Supplementary file 1 [file genes-15-00771-s001.zip › Figure S2.pdf]

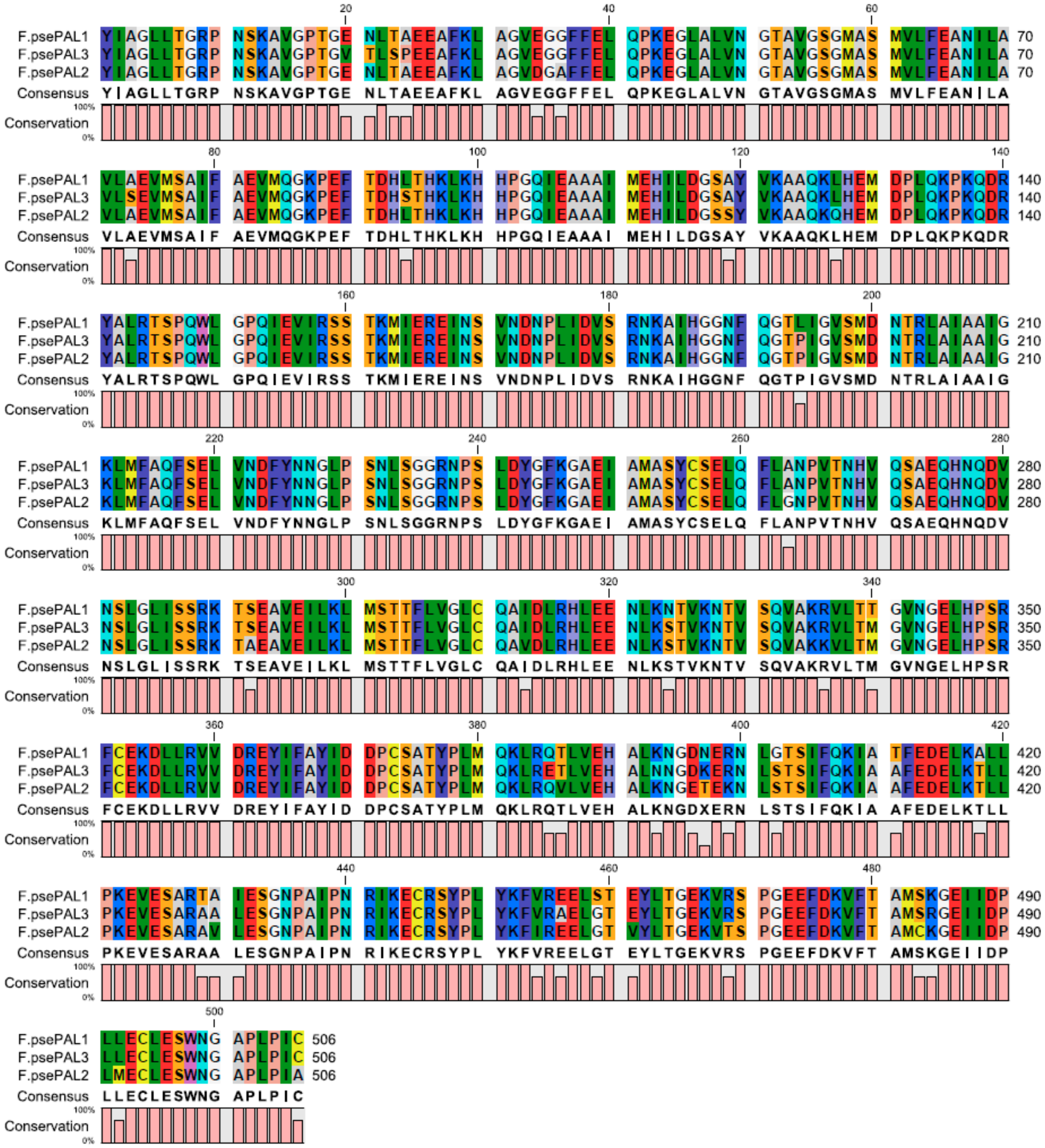

Figure S2. Protein sequences alignment of PAL1, PAL2, and PAL3 genes from *Ferula pseudalliacea*. In the Consensus sequence, the letter N indicates a variation in each of the three sequences.
